# Down‐regulation of diesel particulate matter‐induced airway inflammation by the PDZ motif peptide of ZO‐1

**DOI:** 10.1111/jcmm.15843

**Published:** 2020-09-15

**Authors:** Dong Hee Kang, Tae‐Jin Lee, Ji Wook Kim, Yu Som Shin, Ju Deok Kim, Sung Won Ryu, Siejeong Ryu, Yung Hyun Choi, Cheol Hong Kim, EunAe You, SangMyung Rhee, Kyoung Seob Song

**Affiliations:** ^1^ Department of Anesthesiology and Pain Medicine Kosin University College of Medicine Busan Korea; ^2^ Department of Anatomy College of Medicine Yeungnam University Daegu Korea; ^3^ Department of Biochemistry College of Korean Medicine Dong‐Eui University Busan Korea; ^4^ Department of Pediatrics Sungkyunkwan University Samsung Changwon Hospital Changwon Korea; ^5^ Department of Life Science Chung‐Ang University Seoul Korea; ^6^ Department of Cell Biology Kosin University College of Medicine Busan Korea

**Keywords:** airway inflammation, PDZ domain, PM2.5, RGS12, ZO‐1

## Abstract

Although diesel airborne particulate matter (PM2.5) has been known to play a role in many human diseases, there is no direct evidence that therapeutic drugs or proteins can diminish PM2.5‐induced diseases. Nevertheless, studies examining the negative control mechanisms of PM2.5‐induced diseases are critical to develop novel therapeutic medications. In this study, the consensus PDZ peptide of ZO‐1 inhibited PM2.5‐induced inflammatory cell infiltration, pro‐inflammatory cytokine gene expression, and TEER in bronchoalveolar lavage (BAL) fluid and AM cells. Our data indicated that the PDZ domain in ZO‐1 is critical for regulation of the PM2.5‐induced inflammatory microenvironment. Therefore, the PDZ peptide may be a potential therapeutic candidate during PM‐induced respiratory diseases.

## 1 INTRODUCTION

Air pollution is a global concern and is becoming increasingly severe in Korea. The annual average airborne 2.5‐µm particulate matter (PM2.5) concentration in Korea was 24.0 µg/m^3^ in 2018, but was only 12.0 µg/m^3^ in Japan, 9.0 µg/m^3^ in USA, and 7.9 µg/m^3^ in Canada. The PM2.5 concentration in Korea is 2.4‐fold greater than the 10 µg/m^3^ annual guideline for PM2.5 recommended by the World Health Organization, and approaches the recommended 25 µg/m^3^ 24‐hour mean.^1^ The best understanding of molecular mechanisms that contribute to the physiological functions of tight junction protein may be critical for establishing new therapeutic medications for PM2.5‐induced airway inflammation. ZO‐1 was affected by pro‐inflammatory cytokines and the tight junctional barrier of the upper and lower respiratory tract, as assessed by TEER testing, was reduced. The ZO‐1 protein is composed of three PSD95/dlg/ZO‐1 (PDZ) domains, an SH3 domain, and a region of homology to guanylate kinase (GUK)^2^; however, there have been few studies of the effects of PDZ of ZO‐1 on PM‐induced airway inflammation.

## 2 MATERIALS

The National Institute of Standards and Technology (NITS, Gai‐thersburg, MD, USA) SRM 1650b standard diesel PM (PM2.5), with diameters below 2.5 mm, was purchased from MERCK (NIST‐1650b).^3^ PDZ peptides were synthesized by Peptron. The human bronchial epithelial cell line (BEAS‐2B; CRL‐9609)) and human alveolar basal epithelial cell line (A549; CCL‐185) were purchased from the American Type Culture Collection. BEAS‐2B was cultured in BEGM™ Bronchial Epithelial Cell Growth Medium BulletKit™ (Lonza; CC‐3170).

## 3 RESULTS

**FIGURE 1:**
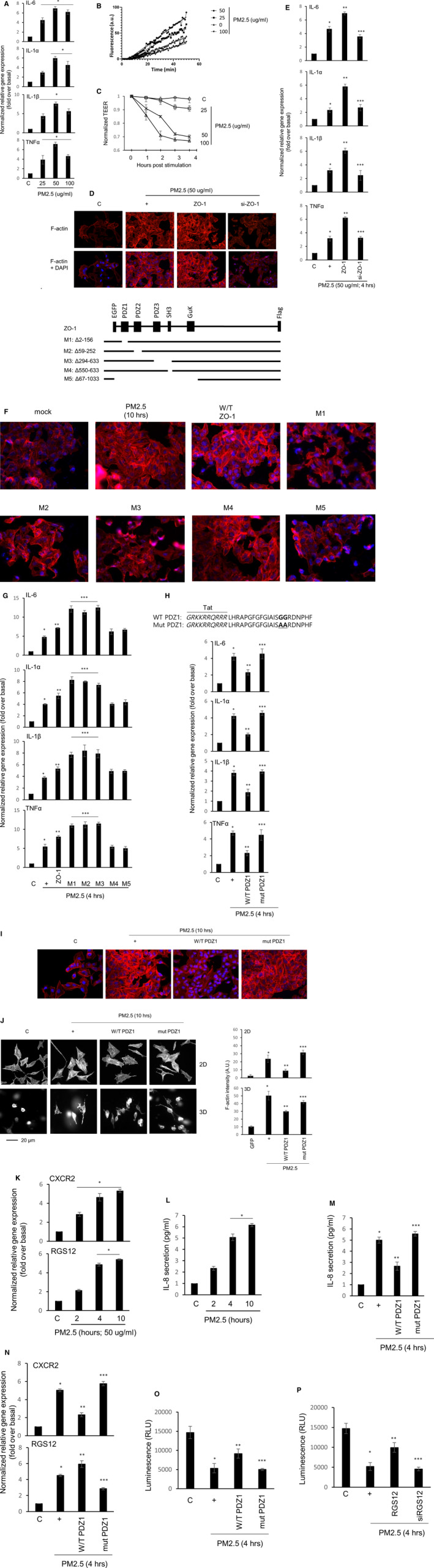
Effect of PDZ domain of ZO‐1 protein on PM2.5‐induced airway inflammation. A, Quiescent BEAS‐2B cells were incubated for 4 h with various concentrations of PM2.5, and then lysates were harvested and analyzed by real‐time quantitative RT‐PCR. *P < .05 compared to the control. β_2_M, beta‐2‐microglobulin, was used as a loading control. B, The effects of PDZ peptides on actin polymerization were examined using an Actin Polymerization Biochmem Kit™ (BK003, Cytoskeleton). The cells were treated with PM2.5 for 4 h, and cell lysates were collected, centrifuged, and then supernatant recovered. Cell lysates with equal amounts of protein were treated with the final reaction mix containing ATP and pyrene‐conjugated actin (final concentration = 0.4 mg/mL) in actin polymerization buffer. Actin polymerization was visualized by fluorescence intensity using a microplate reader with a 355‐nm excitation filter and a 405‐nm emission filter, and the analyses were performed using Microsoft Excel. C, The cells were treated with PM2.5 for 4 h, and the TEER was measured. Error bars represent the SEM of at least three independent experiments. D, A549 cells were transfected with either a construct driving the expression of wild‐type ZO‐1 or ZO‐1‐specific siRNA. Cells were then incubated with PM2.5 for 10 h. The cells were stained with ActinGreen™ 488 ReadyProbe reagent (R37112, Molecular Probes). E, Cells were transfected, and were then incubated with PM2.5 for 4 h prior to the generation of total cell lysates, and then qRT‐PCR for pro‐inflammatory cytokines transcript was performed.^14^ *P < .05 compared to the control, **P < .05 compared to PM2.5 only, and ***P < .05 compared to ZO‐1‐transfected cells. F, Constructs were designed according to the amino acids deleted (*eg* M1: 2‐156). Black lines represent the magnitude of the sequences encoded by each construct. The cells were transfected with either a ZO‐1 overexpression construct or deletion constructs, and were incubated with PM2.5 for 10 h. F‐actin was stained with a specific reagent. G, Cells were transfected with deletion constructs and were then incubated with PM2.5 for 4 h, after which qRT‐PCR for pro‐inflammatory cytokine transcripts was performed. *P < .05 compared with the control, **P < .05 compared with PM2.5 only, and ***P < .05 compared with ZO‐1‐transfected cells. H, Peptides were synthesized with Tat region (italic amino acids) based on the first PDZ domain sequence (upper panel). Cells were treated with consensus PDZ or GG25,26AA mutant PDZ peptide for 24 h and then incubated with PM2.5 for either 4 or 10 h (I), after which we performed qRT‐PCR and F‐actin staining. *P < .05 compared with the control; **P < .05 compared with PM2.5 alone; ***P < .05 compared with consensus PDZ peptide treatment. J, Cells were seeded on coverslips and then treated with either consensus PDZ peptide or the mutant PDZ peptide (both at 1.0 µg/mL) prior to treatment with PM2.5. After fixation, rhodamine‐conjugated phalloidin was added for 30 min (1:100 dilution). Cells were then stained with DAPI for 2 min (1:10 000 dilution) (2D culture; upper panels). After trypsinizing, collagen was added to the cells (1.5 mg/mL; 2 × 10^4^/matrix), and subsequently, media containing FBS and PM2.5 was added and the preparations incubated for 10 h. After blocking with 2% BSA, the cells were incubated with rhodamine‐conjugated phallodin (3D culture; lower panels). The scale bar is 20 µm. The F‐actin intensity was used to assess morphometric differences between cells. *P < .05 compared with the control (GFP); ** *P* < .05 compared with PM2.5 only; ***P < .05 compared with PM2.5 and consensus PDZ peptide treatment. K, Confluent and quiescent cells were incubated for various durations with PM2.5, the lysates and medium were harvested, and then analyzed by real‐time quantitative RT‐PCR and IL‐8‐specific ELISA (L). *P < .05 compared with the control. M, Cells were treated with consensus PDZ or mutant PDZ peptide for 24 h and then incubated with PM2.5 for 4 h. An IL‐8 was assayed in the medium using ELISA and qRT‐PCR (N) was carried out with cell lysates. *P < .05 compared with the control; **P < .05 compared with PM2.5 only; ***P < .05 compared with PM2.5 and consensus PDZ peptide treatment. O, The cells were treated with consensus PDZ or mutant PDZ peptide. After 24 h, the cells were re‐trypsinized, and seeded at 7000 cells/well into a 96‐well plate. The cells were incubated with PM2.5 for 4 h, and a cAMP assay was performed according to the manufacturer's instructions (cAMP‐Glo assay; Promega). *P < .05 compared with the control; **P < .05 compared with PM2.5 only; ***P < .05 compared with PM2.5 and consensus PDZ peptide treatment. P, The cells were transfected with an RGS12 overexpression construct or siRNA‐RGS12, the cells were re‐trypsinized, and seeded at 7000 cells/well into a 96‐well plate. The cells were incubated with PM2.5 for 4 h, and a cAMP assay was performed. *P < .05 compared with the control; **P < .05 compared with PM2.5 only; ***P < .05 compared with PM2.5 and RGS12 treatment. All of data shown are representative of three independent experiments

PM2.5 at 25 ~ 75 µg/mL did not have any cytotoxic effects on BEAS‐2B cells. To determine whether PM2.5 could induce airway inflammation in the airway cells, we assessed inflammatory cytokines in the cell cultures using qPCR. PM2.5 dramatically induced *IL‐6*, *IL‐1α*, *IL‐1β*, and *TNFα* gene expression in BEAS‐2B cells (Figure 1A). In addition, actin formation was increased in a PM2.5 concentration‐dependent manner, whereas TEER was reduced in a dose‐dependent manner (Figure 1B,C). Unfortunately, because BEAS‐2b cells have several technical limitations, like transfection and growth. We used the A549 line as it imitates all the major characteristics of primary alveolar type II (ATII) cells, allowing collection of consistent and reproducible data without the technical or ethical issues involved in using primary or stem cells.^4^ Whereas ZO‐1 overexpression increased the density of PM2.5‐induced F‐actin formation, siRNA‐ZO‐1 did not (Figure 1D). ZO‐1 overexpression increased pro‐inflammatory cytokines gene expression, but si‐RNA‐ZO‐1 significantly reduced their expression (Figure 1E). The reasons why overexpression of ZO‐1 increased pro‐inflammatory cytokine gene expression: First, because PM2.5 releases/produces inflammatory cytokines to induce airway inflammation, ZO‐1 expression increased in proportion to the degree of inflammation in the acute phase, and then ZO‐1 negatively regulated pro‐inflammatory cytokine gene expression. Next, to examine which domain of ZO‐1 was critical for the functions during PM2.5 exposure, several deletion mutants were generated (Figure 1F).^2^ PDZ deletion mutants abolished F‐actin formation, but an SH3 deletion mutant construct did not. In addition, *pro‐inflammatory cytokines* gene expression was significantly increased in the cells transfected with PDZ deletion mutants (M1‐3; Figure 1G). To know the functions of PDZ domain, we synthesized permeable PDZ‐containing specific peptide and mutant peptide (Figure 1H). The consensus PDZ peptide significantly reduced *pro‐inflammatory cytokines* gene expression and F‐actin polymerization, but the mutant PDZ peptide did not (Figure 1H,I). On either 2D (upper panel) or 3D (lower panel) collagen‐coated coverslips,^5^ the formation of F‐actin was enhanced by PM2.5. Whereas the consensus PDZ peptide significantly reduced F‐actin polymerization, the mutant peptide increased (Figure 1J). Next, we investigated the physiological mechanism by which PM2.5 activates pro‐inflammatory cytokine gene expression. PM2.5 may increase *C‐X‐C motif chemokine receptor* (*CXCR*) *2* and *regulator of G protein signaling 12* (*RGS12*) gene expression in a time‐dependent manner (Figure 1K). Intracellular CXCR2 ligand, IL‐8, was secreted from the cells into the medium (Figure 1L). Consensus PDZ peptide inhibited IL‐8 secretion, but mutant peptide did not (Figure 1M). In addition, *CXCR2* gene expression was reduced by the consensus PDZ peptide, but mutant peptide increased. The PDZ domain was localized at the N‐terminal region of RGS12 and RGS12 bound to CXCR2.^6^ Consensus PDZ peptide increased *RGS12* gene expression, rather than reducing *CXCR2* (Figure 1N). Because CXCR2 is a Gαi‐coupled receptor, cAMP concentrations were strongly restored by consensus PDZ peptide, but not by mutant PDZ peptide (Figure 1O). RGS12 also restored cAMP concentrations (Figure 1P). These results indicate that the consensus PDZ peptide acts as a primary‐negative regulator to maintain homeostasis during PM2.5‐induced airway inflammation and RGS12 acts as a secondary negative regulator protein.

**FIGURE 2:**
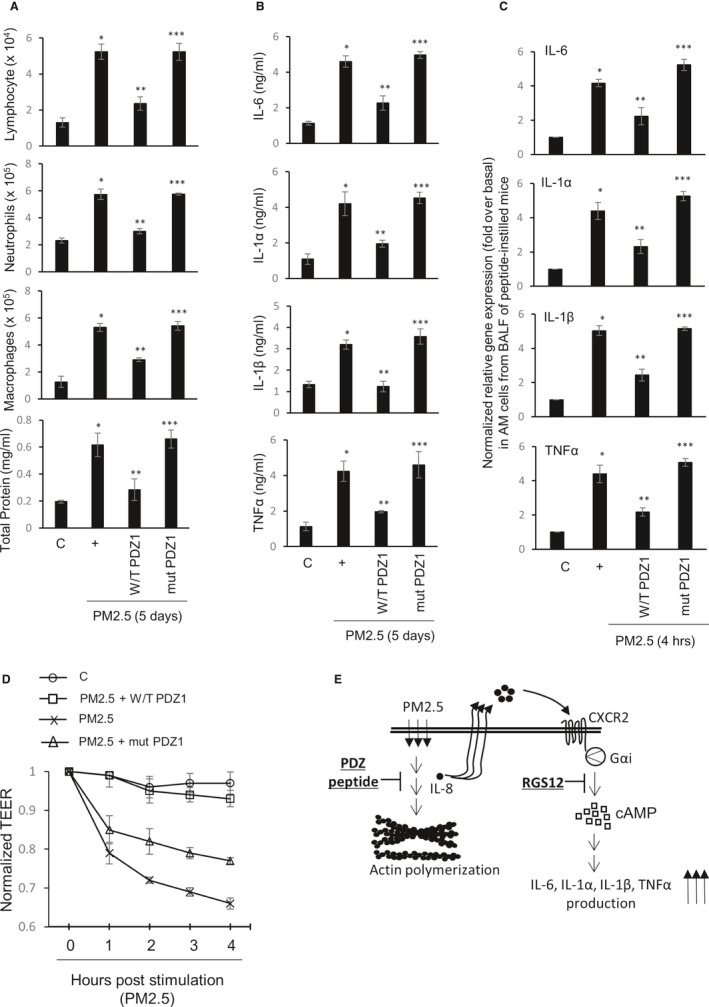
Effect of PDZ peptide on PM2.5‐induced lung inflammatory responses in vivo. A, Five days after PM2.5 instillation (30 µL of 20 mg/kg) into the tracheal lumens of mice that had been injected with either the consensus PDZ peptide or the mutant peptide (2.0 mg/kg/30 µL) 24 h previously. The lymphocytes, neutrophils, alveolar macrophages (AMs), and total protein in the BAL fluid were then assessed. B, The IL‐6, IL‐1α, IL‐1β, and TNFα concentrations in the BAL fluid were measured using specific ELISAs. C, AMs from the BAL fluid in healthy mice were treated with either the consensus PDZ peptide or the mutant peptide prior to incubation with PM2.5 for 4 h, and then, qRT‐PCR was performed. *P < .05 compared with saline‐treated mice; **P < .05 compared with PM2.5‐treated mice; ***P < .05 compared with PM2.5‐ and consensus PDZ peptide‐treated mice. D, The AMs from the BAL fluid in healthy mice were treated with either the consensus PDZ peptide or the mutant peptide prior to incubation with PM2.5 for various times, and then TEER testing was performed. Error bars represent the SEM of at least three independent experiments. All of data shown are representative of three independent experiments

Next, to investigate whether the consensus PDZ peptide regulates the inflammatory cell populations in BAL fluid, we measured various inflammatory cell populations after intranasal instillation of the two PDZ peptides but prior to PM2.5 instillation (Figure 2A). Mice instilled with consensus PDZ peptide had lower levels of inflammatory cells, but mutant peptide increased them. Treatment with the consensus PDZ peptide dramatically reduced PM2.5‐induced pro‐inflammatory cytokine production, whereas mutant PDZ peptide increased (Figure 2B). Consensus PDZ peptide also reduced pro‐inflammatory cytokine gene expression in the lung, whereas mutant peptide increased their expression (Figure 2C). Lastly, TEER was also restored by consensus PDZ peptide, but not by mutant peptide (Figure 2D). Our results indicate that the consensus PDZ peptide abolishes changes in inflammatory cell populations, and pro‐inflammatory cytokine production in both BAL fluid and the lung after PM2.5 treatment.

## 4 DISCUSSION

ZO‐1 is a scaffolding protein that connects transmembrane tight junctions with cytoplasmic proteins and the actin cytoskeleton.^2^, ^7^ The PDZ domain forms dimers or binds to intracellular proteins and PDZ‐containing proteins.^8^, ^9^ The PDZ domain is involved in intracellular signaling, cell adhesion, ion transport, and formation of the paracellular barriers.^10^, ^11^ Unlike the PDZ domain, SH3 and GUK domains did not affect in our system. The functionality of each PDZ domain did not vary and deletion of each domain abolished (Figure 1F,G). Interestingly, the classical PDZ domain at the N‐terminus of RGS12 bound selectively to C‐terminal (A/S)‐T‐X‐(L/V) motifs as found within both the CXCR2 IL‐8 receptor, and the alternative 3′ exon form of RGS12,^6^, ^12^ suggesting that this interaction shows a specific mechanism by which RGS12 acts as a desensitization protein that shuts down GPCR signaling (Figure 2E).^13^

PDZ acts as a negative regulator to maintain homeostasis by shutting down PM2.5‐induced effects in vivo and AMs. Thus, these results suggest that consensus PDZ peptide reduces PM2.5‐mediated F‐actin polymerization, consistent with the observation that the PDZ peptide negatively regulates chemokine‐induced movement to control inflammation at the inflamed site and PDZ peptide may be a potential therapeutic candidate during PM‐induced respiratory diseases.

## CONFLICT OF INTEREST

The authors confirm that there are no conflicts of interest.

## AUTHOR CONTRIBUTIONS

**Dong Hee Kang:** Conceptualization (lead); Data curation (lead); Writing‐original draft (lead). **Tae‐Jin Lee:** Conceptualization (lead); Methodology (lead); Writing‐original draft (lead). **Ji Wook Kim:** Methodology (equal). **Yu Som Shin:** Methodology (equal). **Ju Deok Kim:** Methodology (equal). **Sung Won Ryu:** Methodology (equal). **Siejeong Ryu:** Methodology (equal); Supervision (equal). **Yung Hyun Choi:** Methodology (equal); Supervision (equal). **Cheol Hong Kim:** Data curation (equal); Validation (equal). **EunAe You:** Methodology (equal). **SangMyung Rhee:** Methodology (equal). **Kyoung Seob Song:** Conceptualization (lead); Writing‐review & editing (lead).

## Data Availability

The data sets used and/or analyzed during the current study are available from the corresponding author on reasonable request.
